# Measuring self-reported HIV status in bio-behavioural surveys

**DOI:** 10.2471/BLT.15.153064

**Published:** 2015-05-01

**Authors:** Lisa G Johnston, Willi McFarland, Miriam Lewis Sabin, Dimitri Prybylski, Keith Sabin, Stefan Baral, Andrea A Kim, H Fisher Raymond

**Affiliations:** a Global Health Sciences, University of California-San Francisco, 550 – 16th Street (3rd floor), San Francisco, CA 94158, United States of America (USA).; b San Francisco Department of Public Health, San Francisco, USA.; c The Global Fund to Fight AIDS, Tuberculosis and Malaria, Geneva, Switzerland.; d US Centers for Disease Control and Prevention, Global AIDS Program Asia Regional Office, Nonthaburi, Thailand.; e Joint United Nations Programme on HIV/AIDS, Geneva, Switzerland.; f Center for Public Health and Human Rights, Department of Epidemiology, Johns Hopkins School of Public Health, Baltimore, USA.; g US Centers for Disease Control and Prevention, Division of Global HIV/AIDS, Atlanta, USA.

Currently, many epidemiological and surveillance surveys of general and key populations do not ask participants the results of their most recent test for human immunodeficiency virus (HIV) test. Not asking about HIV status precludes measuring the cascade of engagement in HIV-related care thus undermining the ability to track key indicators in the response to the epidemic. Common reasons cited why participants are not asked their current HIV status in surveys include: (i) doing so may violate respondents’ human rights; (ii) asking has the potential to exacerbate stigma and discrimination; and (iii) self-reported status can be inaccurate. A crisis caused by potential and real violations of privacy led to public health surveillance that collects less health information compared to other infections. “AIDS exceptionalism”, once necessary in settings with punitive laws and human rights violations, has carried forward from the 1980s to today.^1^

Changes in social and biomedical contexts over the past decade diminish the need for an exceptional approach to HIV surveillance. The availability of life-prolonging antiretroviral therapy (ART) and the communal benefit of reduced onward HIV transmission provide a strong rationale for early detection of HIV infection.^2^ Stigma and discrimination caused by asking survey participants their HIV status, in addition to other routinely posed sensitive questions (e.g. condom use, arrests, sharing needles/syringes and forced sex) can be minimized in several ways. All surveys include confidentiality and anonymity for participants, ethics review of protocols and strong staff training to maintain confidentiality and anonymity, with clear penalties for disclosing confidential information. Informed consent procedures must always allow participants the right to refuse to respond to questions they find too sensitive or stigmatizing and to discontinue at any time without penalty.

We assert that the benefits of asking self-reported HIV status as part of an HIV survey now outweigh the risks of asking this question. There can be immediate benefits to respondents who can be referred to appropriate services. Public health benefits include information about undiagnosed infection and whether an individual is in HIV-related care and treatment. This is crucial to understanding the potential for HIV epidemic expansion in a given community and coverage of care and treatment services.^3^

Omitting HIV status represents a missed opportunity to measure the undiagnosed proportion of people living with HIV, their treatment coverage and subsequent prevention impact. To estimate the true prevalence of HIV, estimates of the proportion of HIV that is undiagnosed are needed.^4^ Reducing the number of people unaware of their HIV status is a UNAIDS global target precisely because this knowledge is critical to entry into treatment.^5^ Behavioural risk factors for HIV infection may differ among people with unrecognized HIV compared to those with confirmed HIV. Information from people with unrecognized HIV has the potential to improve HIV programme planning and implementation. Mistakenly thinking one is uninfected can lead to incorrect use of harm reduction strategies in which behaviour is adapted according to the assumed HIV status of sexual partners.^6^ Some HIV interventions depend upon awareness of HIV status (e.g. early treatment for couples in which only one partner is infected).^7^ Information on the population groups most likely to have HIV but not aware of their status can be used to prioritize and tailor intervention programmes and allocate resources to those most in need.^8^

Finally, data on status can be used to monitor HIV testing uptake and efficiency.^9^ HIV surveys should routinely ask for self-reported HIV status to characterize the state of the HIV epidemic response. Normalizing reporting of HIV status is one step in a move from exceptional HIV surveillance to standard infectious disease surveillance. In this era of early treatment eligibility and access, with survey test results increasingly being returned to respondents,^10^ asking HIV status can be seen as an ethical imperative, to permit correct referrals, while being minimally intrusive. While stigma and discrimination remain important concerns and require appropriate safeguards, we believe that asking about HIV status can lead to a more robust response to HIV control and prevention.

## References

[1] Bayer R, Edington C. HIV testing, human rights, and global AIDS policy: exceptionalism and its discontents. J Health Polit Policy Law. 2009 6;34(3):301–23. 10.1016/S1473-3099(13)70329-3 19451406 PMC2730828

[2] Nolan S, Wood E. End of the debate about antiretroviral treatment initiation. Lancet Infect Dis. 2014 4;14(4):258–9. 10.1215/03616878-2009-002 24602843 PMC4084688

[3] Gardner EM, McLees MP, Steiner JF, Del Rio C, Burman WJ. The spectrum of engagement in HIV care and its relevance to test-and-treat strategies for prevention of HIV infection. Clin Infect Dis. 2011 3 15;52(6):793–800. 10.1093/cid/ciq243 21367734 PMC3106261

[4] Raymond HF, Bereknyei S, Berglas N, Hunter J, Ojeda N, McFarland W. Estimating population size, HIV prevalence and HIV incidence among men who have sex with men: a case example of synthesising multiple empirical data sources and methods in San Francisco. Sex Transm Infect. 2013 8;89(5):383–7. 10.1136/sextrans-2012-050675 23620133

[5] Fast track: ending the AIDS epidemic by 2030. Geneva: UNAIDS; 2014.

[6] McFarland W, Chen YH, Nguyen B, Grasso M, Levine D, Stall R, et al. Behavior, intention or chance? A longitudinal study of HIV seroadaptive behaviors, abstinence and condom use. AIDS Behav. 2012 1;16(1):121–31. 10.1007/s10461-011-9936-8 21644001 PMC3686637

[7] Li X, Lu H, Raymond HF, Sun Y, Jia Y, He X, et al. Untested and undiagnosed: barriers to HIV testing among men who have sex with men, Beijing, China. Sex Transm Infect. 2012 4;88(3):187–93. 10.1136/sextrans-2011-050248 22158932

[8] Young SD, Shoptaw S, Weiss RE, Munjas B, Gorbach PM. Predictors of unrecognized HIV infection among poor and ethnic men who have sex with men in Los Angeles. AIDS Behav. 2011 4;15(3):643–9. 10.1007/s10461-009-9653-8 20043200 PMC3029495

[9] Do TD, Chen S, McFarland W, Secura GM, Behel SK, MacKellar DA, et al. HIV testing patterns and unrecognized HIV infection among young Asian and Pacific Islander men who have sex with men in San Francisco. AIDS Educ Prev. 2005 12;17(6):540–54. 10.1521/aeap.2005.17.6.540 16398576

[10] Baggaley R, Johnson C, Garcia Calleja JM, Sabin K, Obermeyer C, Taegtmeyer M, et al. Routine feedback of test results to participants in clinic- and survey-based surveillance of HIV. Bull World Health Organ. 2015;93(5):352–5. 10.2471/BLT.15.153031 PMC4431522 26229207

